# Effect of cognitive behavioral therapy on anxiety and depression in patients with psoriasis: A protocol for systematic review and meta-analysis

**DOI:** 10.1097/MD.0000000000027720

**Published:** 2021-11-19

**Authors:** Chuan Tan, Jianmei Jiang, Xiaoling Deng, Wei Xiang, Tingting Hu

**Affiliations:** The Central Hospital of Enshi Tujia and Miao Autonomous Prefecture, Enshi, Hubei Province, China.

**Keywords:** anxiety, cognitive behavioral therapy, depression, psoriasis

## Abstract

**Background:**

Psoriasis can lead to higher social and psychological burden, and its occurrence is related to psychological disturbance. At present, there are many clinical trials on cognitive behavior therapy for depression and anxiety in patients with psoriasis. However, the results of studies vary greatly due to the different time and content of the intervention, and the curative effect is still controversial. Therefore, the purpose of this study was to explore the effect of cognitive behavior therapy on anxiety and depression in patients with psoriasis through meta-analysis.

**Methods:**

We searched Chinese and English databases to collect all randomized controlled trials of cognitive behavior therapy on anxiety and depression in patients with psoriasis until October 2021. Two researchers then independently screened articles, extracted data, and evaluated the quality of selected literatures. All data were processed by Stata 14.0.

**Results:**

The results will be published in peer-reviewed journals.

**Conclusion:**

Our study is expected to provide high-quality evidence-based medicine advice for the effects of cognitive behavior therapy on anxiety and depression in patients with psoriasis.

**Ethics and dissemination:**

Ethical approval was not required for this study. The systematic review will be published in a peer-reviewed journal, presented at conferences, and shared on social media platforms. This review would be disseminated in a peer-reviewed journal or conference presentations.

**OSF REGISTRATION NUMBER:**

DOI 10.17605/OSF.IO/APKVG.

## 1 Introduction

Psoriasis is a chronic skin disease that is difficult to cure, and it is changeable and easy to occur repeatedly, mostly in young adults and male patients.^[1–3]^ The main symptoms include red papules, erythema, and silver-white scales.^[4–6]^ So far, the cause of psoriasis is not clear.^[7]^ According to related research, there are many inducing factors, including heredity, infection, abnormal immune function, abnormal metabolic function, abnormal endocrine function, and so on.^[8,9]^ Due to the long course of treatment of psoriasis, the appearance of patients is seriously affected at the same time. Patients are easy to be inferior, pessimistic, negative, and even depressed.^[10–13]^

Studies have revealed that many patients with psoriasis experience a wide range of psychological difficulties, thus increasing the risk of depression, anxiety, social phobia, persistent worry, suicidal thoughts, smoking, and alcohol abuse.^[14–18]^ One of the important causes of psychosocial pain in patients with psoriasis lies in low self-esteem caused by negative body image and social withdrawal.^[19–22]^ In addition, patients often lack control over psoriasis outbreaks and feel desperate to cure the disease and deal with symptoms and treatment burdens.^[19]^ Psychosocial distress is often reported as a result of psoriasis as well as a cause of worsening symptoms.^[23,24]^

In addition to drug therapy, psychological intervention is also a necessary treatment for anxiety and depression in psoriasis,^[25,26]^ because psoriasis is related to psychological, physiological, social, and other factors.^[13,27,28]^ The contents of psychological intervention mainly include cognitive behavioral therapy, supportive psychotherapy, music therapy, relaxation training, interest therapy, motivation interview, and so on. Cognitive behavioral therapy refers to correcting patients’ bad mood and behavior by correcting their bad cognition, persuasion, demonstration, relaxation training, behavior training, and so on.^[29]^ Studies at home and abroad have proved that cognitive behavioral therapy can improve depression, anxiety, and other bad emotions in patients with psoriasis.^[30–32]^ Nonetheless, it has not been confirmed by systematic evaluation.

In order to evaluate the intervention effect of cognitive behavioral therapy on psoriatic patients, this study systematically reviewed all the published randomized controlled trials (RCTs) on the intervention effect of cognitive behavioral therapy on anxiety and depression in patients with psoriasis. This study will provide evidence-based medical evidence for cognitive behavioral therapy in the intervention of psoriasis anxiety and depression.

## 2 Methods

### 2.1 Study registration

The protocol of this review was registered in OSF (OSF registration number: DOI 10.17605/OSF.IO/APKVG). It was reported to follow the statement guidelines of preferred reporting items for systematic reviews and meta-analyses protocol.^[33]^

### 2.2 Inclusion criteria for study selection

#### 2.2.1 Types of studies

RCT was enrolled to investigate the effects of cognitive behavior therapy on anxiety and depression in patients with psoriasis. Language is restricted to Chinese and English.

Non-RCTs, cohort studies, case reports, experimental studies, and the data of the included study were missed or incomplete, and duplicate publications were excluded.

#### 2.2.2 Types of participants

Patient who were diagnosed with psoriasis were enrolled. Patients with major organ dysfunction, mental illness, and cognitive impairment were excluded.

#### 2.2.3 Types of interventions

The experimental group was intervened with cognitive behavioral therapy. The control group was treated with routine treatment or nursing or blank control. Other intervention measures in the experimental group and the control group are basically the same.

#### 2.2.4 Types of outcome measures

Outcome indicators included: depression, measured by depression-related scale. It mainly includes Back Depression Inventory (BDI), Hamilton Depression scale (HAMD), and Geriatric Depression Scale-15 (GDS-15). Anxiety, measured by an anxiety related scale. It mainly includes Inventory Beck Anxiety (BAI), Hamilton Anxiety Scale (HAMA), and Penn State Worry Questionnaire (PSWQ).

### 2.3 Data sources

PubMed, Web of Science, Cochrane Library, EMBASE, Wan fang Database, Chinese Scientific Journal Database, China National Knowledge Infrastructure Database, and Chinese Biomedical Literature Database were systematically searched. The time for literature retrieval was set to build the database until October 2021.

### 2.4 Searching strategy

The details of PubMed's search strategies are illustrated in Table 1, including all search terms, while similar search strategies are applied to other electronic databases.

**Table 1:** Search strategy in PubMed database.

Number	Search terms
#1	Psoriasis[MeSH]
#2	Palmoplantaris Pustulosis[Title/Abstract]
#3	Pustular Psoriasis of Palms and Soles[Title/Abstract]
#4	Pustulosis Palmaris et Plantaris[Title/Abstract]
#5	Pustulosis of Palms and Soles[Title/Abstract]
#6	Psoriases[Title/Abstract]
#7	or/1–6
#8	Cognitive Therapy[MeSH]
#9	Behavior Therapy, Cognitive[Title/Abstract]
#10	Psychotherapy, Cognitive[Title/Abstract]
#11	Cognition Therapy[Title/Abstract]
#12	Cognitive Behavior Therapy[Title/Abstract]
#13	Cognitive Behavioral Therapy[Title/Abstract]
#14	Cognitive Psychotherapy[Title/Abstract]
#15	Therapy, Cognition[Title/Abstract]
#16	Therapy, Cognitive[Title/Abstract]
#17	Therapy, Cognitive Behavior[Title/Abstract]
#18	Behavior Therapies, Cognitive[Title/Abstract]
#19	Behavioral Therapies, Cognitive[Title/Abstract]
#20	Behavioral Therapy, Cognitive[Title/Abstract]
#21	Cognition Therapies[Title/Abstract]
#22	Cognitive Behavior Therapies[Title/Abstract]
#23	Cognitive Behavioral Therapies[Title/Abstract]
#24	Cognitive Psychotherapies[Title/Abstract]
#25	Cognitive Therapies[Title/Abstract]
#26	Psychotherapies, Cognitive[Title/Abstract]
#27	Therapies, Cognition[Title/Abstract]
#28	Therapies, Cognitive[Title/Abstract]
#29	Therapies, Cognitive Behavior[Title/Abstract]
#30	Therapies, Cognitive Behavioral[Title/Abstract]
#31	Therapy, Cognitive Behavioral[Title/Abstract]
#32	or/8–31
#33	#7 and #32

### 2.5 Data collection and analysis

#### 2.5.1 Literature screening and data extraction

**Figure 1:**
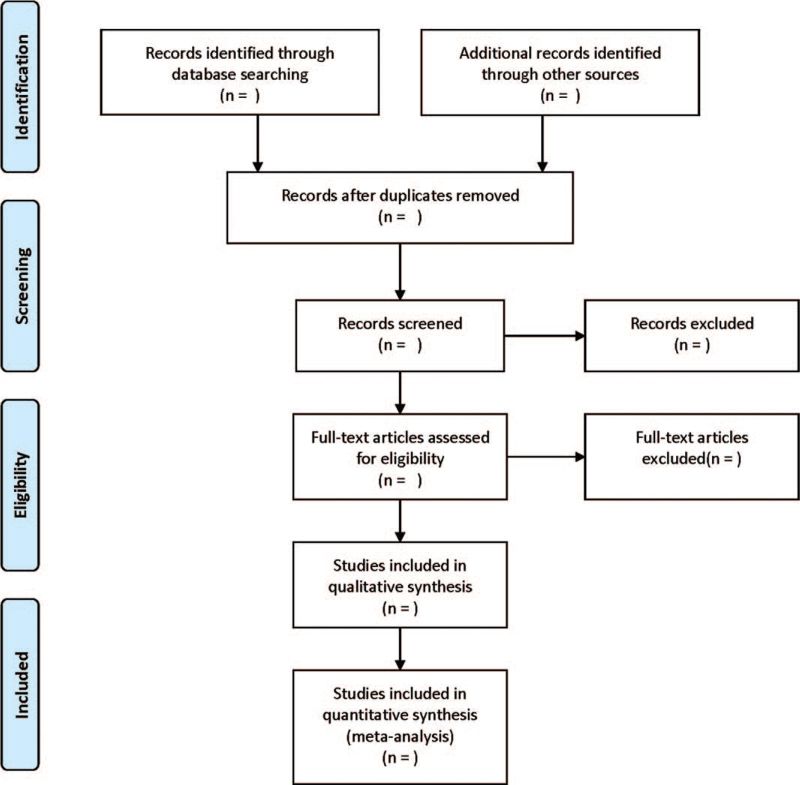
Flow diagram of study selection process.

According to the inclusion and exclusion criteria, 2 researchers independently completed the literature screening. By reading the full text, the data were extracted, and the final results were cross-checked. If there are different opinions, it would be further negotiated and arbitrated with a third researcher. The extraction contents are as follows: basic information such as the year of publication, the place of literature, country, author, etc are included in the study. Research methods include random method, sample size, research object, and blind method selection. Types of cognitive behavioral therapy. Related outcome indicators. The screening flow chart of this study is demonstrated in Fig. 1.

#### 2.5.2 Assessment of risk of bias

According to the bias risk assessment tool recommended by Cochrane Handbook by 2 researchers, literatures were evaluated. The contents of the evaluation include: whether the generation of the random sequence is rigorous, whether the distribution scheme is hidden, what is the implementation of the blind method, whether the results are selectively reported, whether the outcome data is complete and other possible sources of bias (such as testing the outcome evaluation), and whether the 2 groups of baselines are balanced. The degree of bias wind risk is expressed with “yes,” “no,” and “unclear.” The evaluation grade is divided into A level, B level, and C level. Each of the included studies is rated as Grade A. Some of the above criteria are rated as Grade B. If the above criteria are not met, they are rated as Grade C.

#### 2.5.3 Measures of treatment effect

We applied standardized mean differences (SMD) as the studies by conducing different measures to assess the outcomes. The standardized mean differences allow comparison regardless of the used outcome measure.

#### 2.5.4 Management of missing data

If any data is missing, the original data would be requested by email. If the missing data cannot be obtained, the data could be excluded from the study.

#### 2.5.5 Assessment of heterogeneity and data synthesis

Stata 14.0 software (Stata Corp, College Station, TX) was used for statistical analysis. Heterogeneity test: Q test was applied to qualitatively determine inter-study heterogeneity. If *P* ≥ .1, there is no inter-study heterogeneity, while if *P* < .1, there is inter-study heterogeneity. Meanwhile, *I*^2^ value was adopted to quantitatively evaluate the inter-study heterogeneity. As long as *I*^2^ ≤ 50%, the heterogeneity is considered to be good, and the fixed-effect model would be adopted. Provided that *I*^2^ > 50%, it indicates significant heterogeneity, and the source of heterogeneity would be explored through subgroup analysis or sensitivity analysis. On condition that there is no obvious clinical or methodological heterogeneity, it would be considered as statistical heterogeneity, and the random-effect model would be adopted for analysis. If there is significant clinical heterogeneity between the 2 groups, descriptive analysis would be carried out, while subgroup analysis is not required.

#### 2.5.6 Assessment of reporting biases

The funnel plots would be used to examine the publication bias if there are >10 eligible studies.

#### 2.5.7 Subgroup analysis

According to the type of cognitive behavioral therapy, the time of intervention and the severity of the disease, subgroup analysis was conducted.

#### 2.5.8 Sensitivity analysis

Through the study of large weight of elimination effect, the sensitivity analysis was performed to test the stability of the results of meta-analysis.

#### 2.5.9 Grading the quality of evidence

We adopted the Grading of Recommendations Assessment, Development and Evaluation (GRADE) to evaluate the quality of evidence from the following 5 aspects: risk of bias, indirectness, inconsistency, imprecision, and publication bias.^[34]^

#### 2.5.10 Ethics and dissemination

The content of this article does not involve moral approval or ethical review and would be presented in print or at relevant conferences.

## 3 Discussion

Psoriasis is a chronic skin disease that is difficult to cure, and seriously affects the appearance of patients and their life quality,^[35,36]^ and reduces their psychological self-confidence. Active nursing intervention must be taken for patients with psoriasis, so as to improve their confidence and compliance in treatment. Behavioral therapy promotes the improvement of individual cognition and emotion through behavioral changes.^[29]^

In this study, we designed a systematic evaluation scheme based on the latest data, so as to test the effects of cognitive behavioral therapy on the improvement of anxiety and depression in patients with psoriasis. It is hoped that this study can find a more rigorous medical basis for the improvement of anxiety and depression in patients with psoriasis, so as to provide reference for clinical practice. However, there are still some potential limitations in this study. One is the lack of high-quality, multicenter, and large-sample clinical trials, which affect the authenticity of the evidence. Secondly, different types and time of intervention may lead to significant heterogeneity of results.

Therefore, more rigorous, larger samples and higher quality RCTs should be conducted in future studies to confirm the efficacy of cognitive behavioral therapy in terms of improving anxiety and depression in patients with psoriasis.

## Author contributions

**Conceptualization:** Tingting Hu, Chuan Tan.

**Data curation:** Jianmei Jiang.

**Formal analysis:** Jianmei Jiang.

**Funding acquisition:** Tingting Hu.

**Investigation:** Jianmei Jiang.

**Methodology:** Jianmei Jiang, Xiaoling Deng.

**Project administration:** Tingting Hu.

**Resources:** Xiaoling Deng.

**Software:** Xiaoling Deng.

**Supervision:** Tingting Hu.

**Validation:** Wei Xiang.

**Visualization:** Wei Xiang.

**Writing – original draft:** Tingting Hu, Chuan Tan.

**Writing – review & editing:** Tingting Hu, Chuan Tan.
